# Supplementary material for: Venomix: a simple bioinformatic pipeline for identifying and characterizing toxin gene candidates from transcriptomic data
Source: PeerJ. 2018 Jul 31;6:e5361. doi: 10.7717/peerj.5361 (PMC6074769; doi:10.7717/peerj.5361)
Supplement: Supplemental Information 3 [file peerj-06-5361-s003.gz › FinalOutput_E-20/Snake_venom_metalloproteinase_acutolysin-C_1/finaltree.pdf]

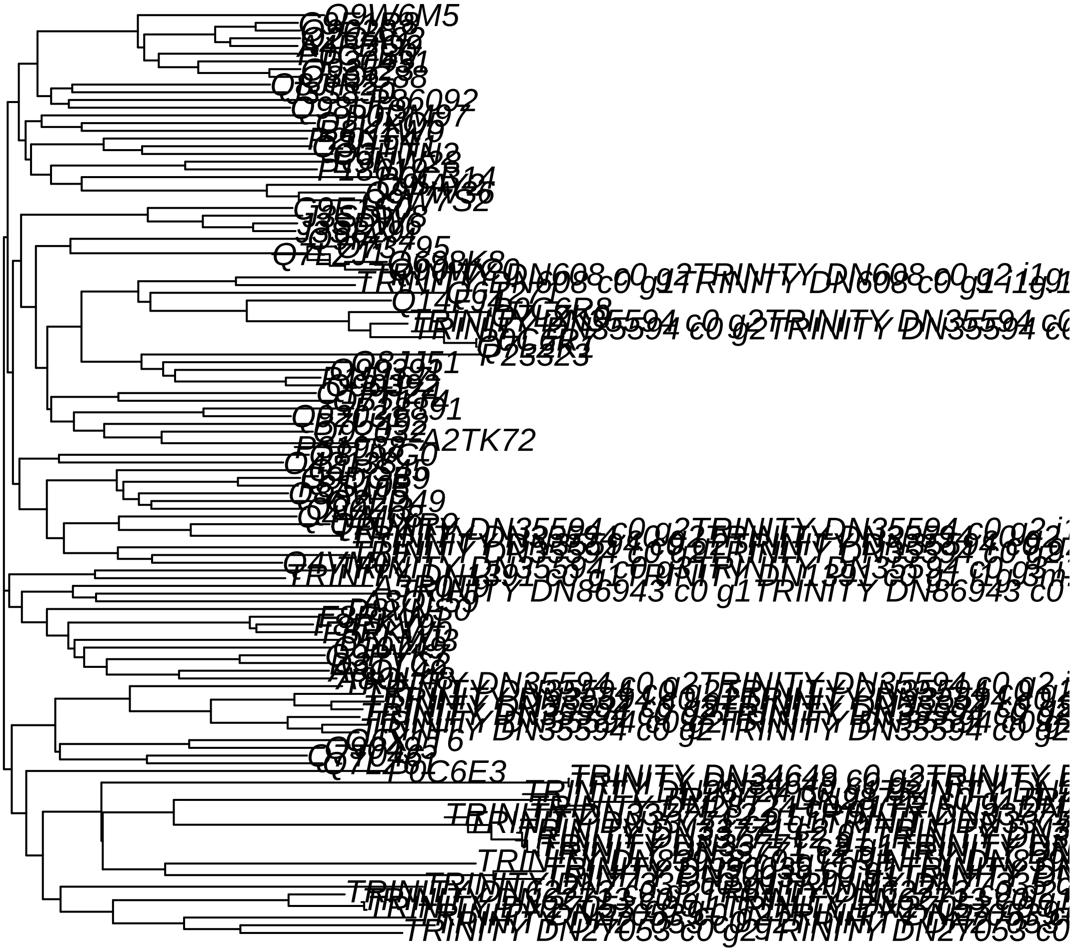
Q9W6M5
TRINITY DN35594 c0 g2TRINITY DN35594 c0
P25323
Q8IJ51
A2TK72
TRINITY DN86943 c0 g1TRINITY DN86943 c0
P0C6E3
TRINITY DN27053 c0 g2TRINITY DN27053 c0
